# Toward a Correct Description of Initial Electronic Coherence in Nonadiabatic Dynamics Simulations

**DOI:** 10.1021/acs.jpclett.4c02418

**Published:** 2024-11-14

**Authors:** Jonathan R. Mannouch, Aaron Kelly

**Affiliations:** Hamburg Center for Ultrafast Imaging, Universität Hamburg and Max Planck Institute for the Structure and Dynamics of Matter, Luruper Chaussee 149, 22761 Hamburg, Germany

## Abstract

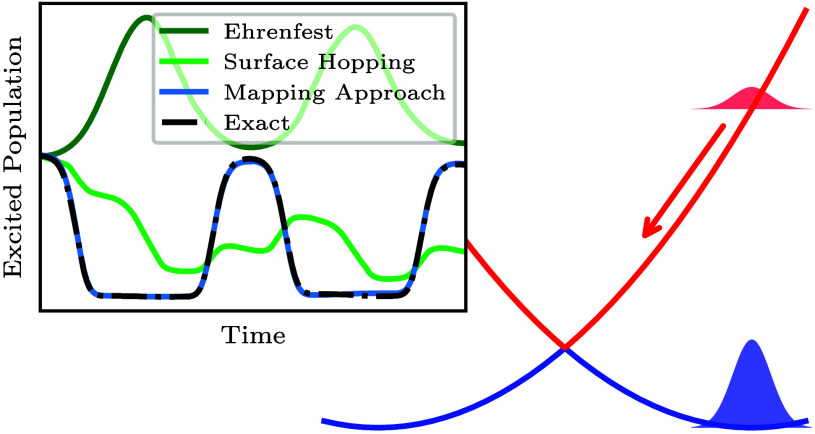

The recent improvement in experimental capabilities for interrogating and controlling molecular systems with ultrafast coherent light sources calls for the development of theoretical approaches that can accurately and efficiently treat electronic coherence. However, the most popular and practical nonadiabatic molecular dynamics techniques, Tully’s fewest-switches surface hopping and Ehrenfest mean-field dynamics, are unable to describe the dynamics proceeding from an initial electronic coherence. While such issues are not encountered with the analogous coupled-trajectory algorithms or numerically exact quantum dynamics methods, applying such techniques necessarily comes with a higher computational cost. Here we show that a correct description of initial electronic coherence can indeed be achieved using independent-trajectory methods derived from the semiclassical mapping formalism. The key is the introduction of an initial sampling over the electronic phase space and a means of incorporating phase interference between trajectories, both of which are naturally achieved when working within the semiclassical mapping framework.

The development and application of coherent light sources for probing and controlling the properties of matter provide substantial motivation for including the effects of the light source in computational simulations.^1^ For example, when simulating ultrafast laser-driven photochemical dynamics, one would like to describe the photoexcitation step on the same footing as the subsequent nonadiabatic relaxation processes. In other words, the molecular system should be initialized in the ground state, and the excitation of the system should be simulated in real time through an explicit description of the pulse. It is, however, known that the most commonly used independent-trajectory approaches for simulating nonadiabatic dynamics in chemistry, Ehrenfest dynamics^2,3^ and fewest-switches surface hopping (FSSH),^4,5^ can fail to capture the correct light-induced coherent dynamics.^6,7^

As a result, most simulations indirectly take the effect of the pulse into account by initializing the system in an incoherent mixture of the photoaccessible excited electronic states,^8,9^ with the nuclei still in their ground-state distribution. This is underpinned by two assumptions, which may or may not be valid in real photochemical scenarios. First, the electromagnetic pulse is assumed to be short on the time scale of the nuclear motion, so that the nuclear wavepacket is not substantially altered by the pulse.^10^ Second, decoherence is assumed to be fast, so that the resulting wavepackets on different electronic surfaces will decohere before any conical intersections are reached.^11^ To go beyond this commonly used computational protocol, a natural first step is to relax the second assumption and initialize simulations in the physically relevant electronic coherence.

Even this simple extension provides a serious challenge for the most commonly used independent-trajectory techniques.^12^ For example, it has been recently shown that Ehrenfest and FSSH cannot describe the initial decoherence of a pair of coherent wavepackets or the subsequent electronic population dynamics on passing through an avoided crossing.^13^ As these methods are perhaps the most practical for treating nonadiabatic dynamics in molecular systems, there is a serious need to develop equally practical methods that can describe this situation correctly.

One possibility is to utilize wave function based approaches that calculate time-evolved observables, , according to
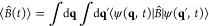
1where |ψ(**q**, *t*)⟩ = *∑*_λ_*c*_λ_(**q**, *t*)|ψ_λ_(**q**)⟩|**q**⟩ is the time-evolved wave function, expressed in terms of an eigenstate of the nuclear position operator, |**q**⟩, and the adiabatic electronic states, |ψ_λ_(**q**)⟩. Such a representation encompasses approximate Gaussian wavepacket techniques^14−17^ and coupled semiclassical-trajectory approaches,^18−21^ derived, for example, via the exact factorization.^22^ A recent paper^13^ has shown that coupled trajectories can indeed alleviate the problems associated with Ehrenfest and FSSH when starting in an initial electronic coherence, albeit at a higher computational cost.

Here we assess the applicability of the semiclassical mapping formalism^23−28^ to this problem. The mapping formalism provides a way of going beyond the standard approaches of Ehrenfest and FSSH, without the need to invoke coupled-trajectory simulation algorithms. Within this formalism, independent-trajectory approaches can be derived by making approximations to real-time correlation functions of the form
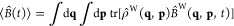
2where  is the partial Wigner transform^29^ of the operator *B̂* with respect to the nuclear degrees of freedom and tr[...] denotes a quantum trace over the electronic degrees of freedom. Additionally, the initial state of the system is expressed in terms of the density matrix, . This provides an additional framework through which nonadiabatic dynamics and decoherence phenomena can be understood.

To better understand the difficulty in describing the dynamics of an initial electronic coherence, it is instructive to first consider what the correct dynamics should look like. In panel 1 of Figure 1, we illustrate a typical photochemical scenario, in which an ultrashort laser pulse has promoted a small fraction of a stable ground-state wavepacket (blue Gaussian) to the excited Born–Oppenheimer electronic surface (red Gaussian). Crucially, because the Born–Oppenheimer surfaces are typically far apart at the Franck–Condon geometry in photochemical systems, the initial dynamics can be decomposed into independent motions associated with each component of the electronic reduced density matrix.^a^ In the classical-nuclear limit, the electronic populations (corresponding to the blue and red Gaussians in Figure 1) evolve on their associated Born–Oppenheimer surface, while the electronic coherences (corresponding to the pink Gaussian in Figure 1) evolve on the average of the surfaces^30−32^ (given by the dashed pink line in Figure 1).

**Figure 1:**
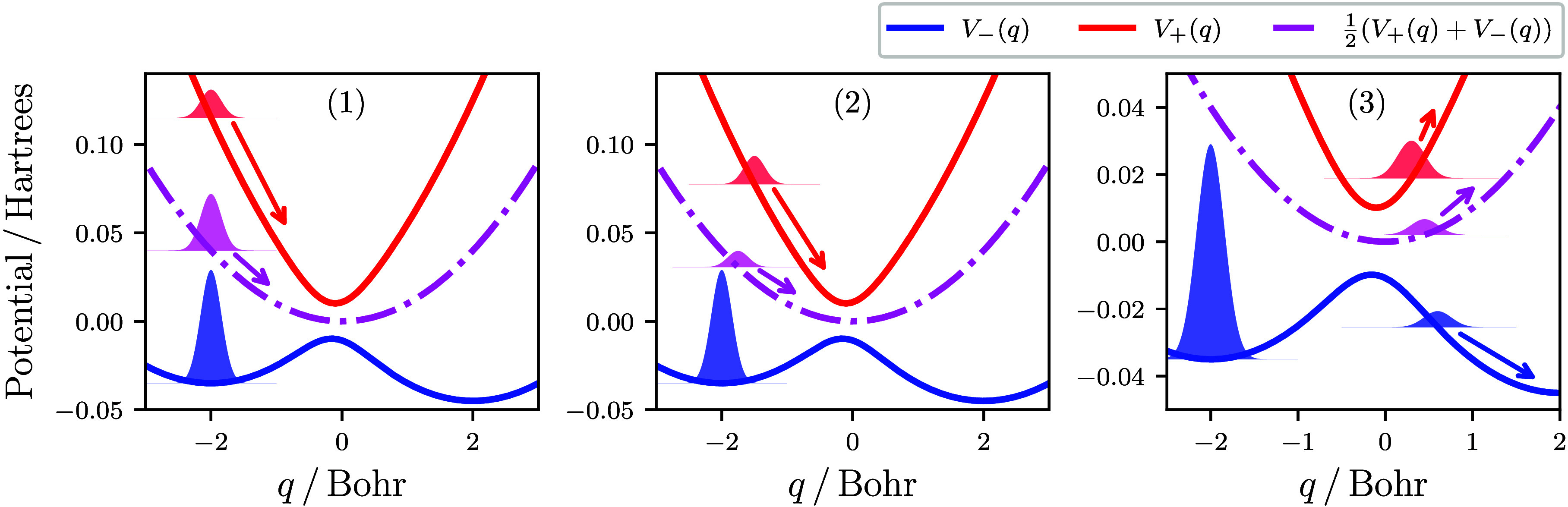
Schematic illustrating certain aspects of the dynamics generated from an initially coherent wavepacket between the ground (blue) and excited (red) Born–Oppenheimer surfaces. The different components of the initial electronic reduced density matrix are represented by the Gaussians in panel 1, with the arrows showing their instantaneous motion. The red and blue Gaussians correspond to the associated adiabatic populations, and the pink Gaussian corresponds to the electronic coherence. Panel 2 illustrates the decoherence of the initially coherent wavepacket, and panel 3 illustrates a nonadiabatic transition, occurring when the excited-state wavepacket passes through the coupling region. The Born–Oppenheimer surfaces in each panel correspond to the same model that is considered in refs (13) and (20).

Applying this to the scenario illustrated in Figure 1, we see that the ground-state population will remain stationary in its potential well, while the excited-state population and the coherences will initially experience a force toward the right. As the excited- and ground-state wavepackets separate, the two will decohere, resulting in a decay of the initial electronic coherence (panel 2 of Figure 1). Later, when the excited-state wavepacket reaches the coupling region at *q* = 0, a nonadiabatic transition will then promote part of this wavepacket onto the ground-state surface, momentarily recreating electronic coherence (panel 3 of Figure 1). Further nonadiabatic transitions occur whenever wavepackets recross the coupling region, which can also lead to “recoherence” events as previously decohered wavepackets on different surfaces re-overlap.

To describe all aspects of these dynamics with independent trajectories, the following two minimal criteria must be satisfied. First, to describe the initial independent motion of the red and blue wavepackets, trajectories must initially feel the force of either the ground- or excited-state surface, with a ratio that matches the associated electronic populations. Second, to describe the initial dynamics of the electronic coherence, some trajectories must also propagate on the average surface.

The problem with Ehrenfest and FSSH is that they do not simultaneously fulfill both of these criteria. While the mean-field force used in Ehrenfest dynamics is suitable for describing the dynamics of the electronic coherence, it fails to correctly propagate the electronic populations on single Born–Oppenheimer surfaces. In contrast, while the surface-hopping force of FSSH guarantees that the electronic populations are correctly propagated on single Born–Oppenheimer surfaces, the electronic coherences are no longer propagated on the average surface. FSSH additionally suffers from a so-called “inconsistency error”,^5^ which arises from the fact that the active propagation surface can become inconsistent with the underlying electronic wave function during the dynamics. While decoherence corrections^33−41^ have been introduced to alleviate the inconsistency error in FSSH, they are not guaranteed to fix the problem.

One way to resolve these issues is to utilize semiclassical mapping approaches. While several different mappings have been suggested,^24,42,43^ in this paper we focus on methods derived within the spin-mapping formalism.^28,44^ This maps a two-state electronic subsystem onto a spin-^1^/_2_ particle and represents any two-state electronic wave function by a spin vector, **S**, on the three-dimensional Bloch sphere. For example, the north and south poles of the Bloch sphere (*S*_*z*_ = ±1, and *S*_*x*_ = *S*_*y*_ = 0) correspond to the excited- and ground-state adiabats, respectively, and the spin vector corresponding to the initial coherent wavepacket shown in panel 1 of Figure 1 is given by the left column of Figure 2.

**Figure 2:**
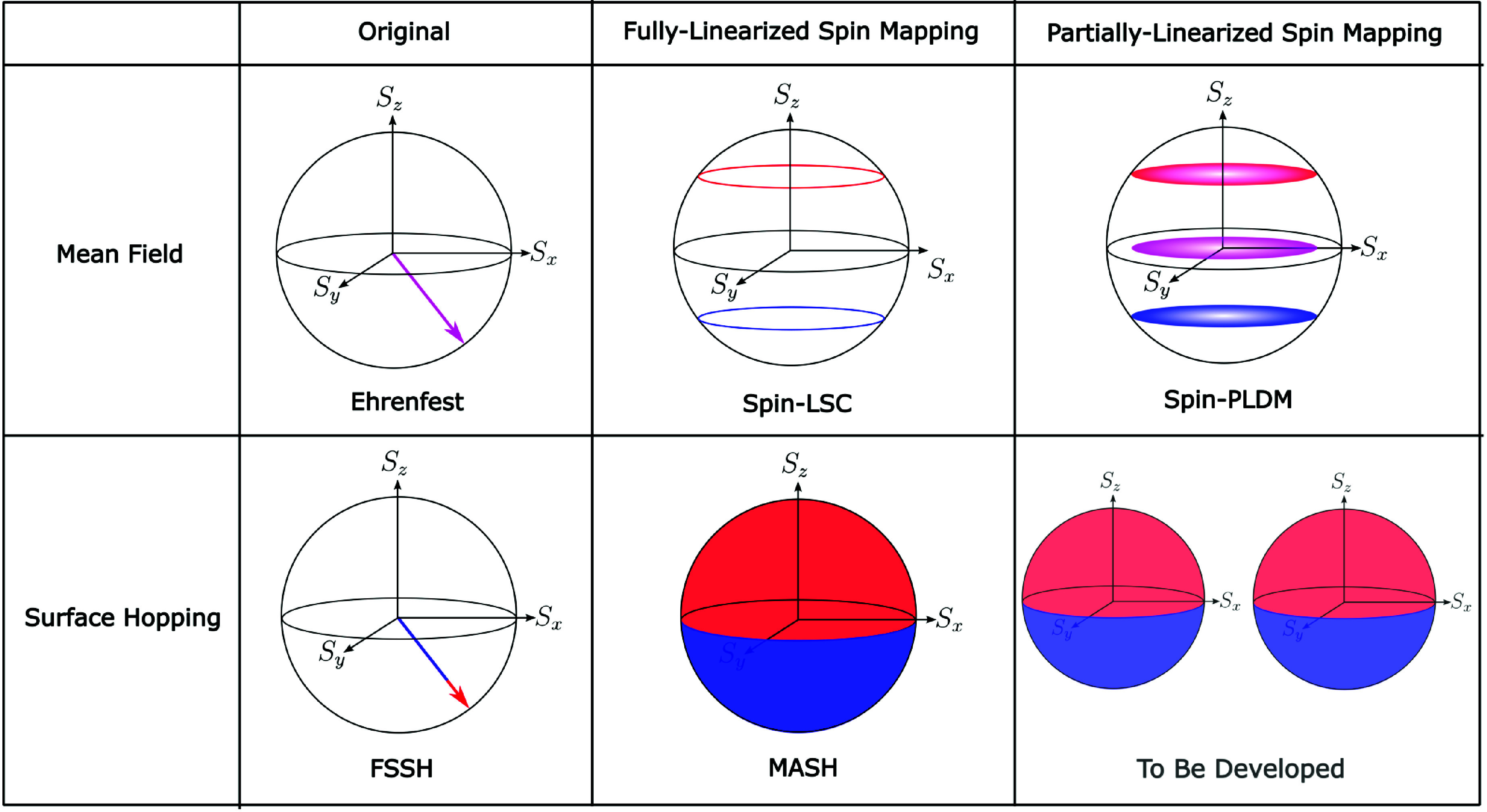
Schematic illustrating the phase-space regions from which the electronic spin-mapping variables are initialized for various independent-trajectory approaches. The red and blue shading signifies regions for which the trajectories would initially feel a force corresponding to the upper and lower adiabatic surfaces, respectively, and the pink shading signifies an initial force that is an average of the two surfaces. For spin-PLDM, the initial distribution of the average of the two sets of spin-mapping variables is given. See the Supporting Information for more details.

As with other mapping approaches, the spin-mapping framework has enabled the development of more accurate independent-trajectory approaches that go beyond Ehrenfest and FSSH.^28,45,46^ We briefly introduce these approaches here, although more details are given in the Supporting Information. Spin mapping was first used to develop a more accurate mean-field approach, called spin-LSC,^28^ which utilizes a larger spin sphere of  to reproduce the correct spin magnitude of a quantum spin-^1^/_2_ particle. On this larger sphere, the spin vectors that represent the two adiabatic states (i.e., ) lie on the two polar circles, as illustrated in the top middle panel of Figure 2. These polar circles constitute the initial sampling regions for the spin-LSC approach. By sampling from the polar circles with the correct weighting, each spin-LSC trajectory has an initial force that corresponds to a single Born–Oppenheimer surface, introducing an essential feature lacking in Ehrenfest dynamics. An initial electronic coherence can also be simultaneously described, because any point on the polar circles generally has a non-zero value for *S*_*x*_ and *S*_*y*_.

Most recently, the spin-mapping framework was used to develop a more accurate surface-hopping approach, called the mapping approach to surface hopping (MASH).^46−48^ The main difference between FSSH and MASH is how the active surface for the propagation of the nuclei is determined. While the active surface in FSSH is switched stochastically according to the time evolution of the underlying electronic wave function, in MASH the active surface is instead chosen according to the spin hemisphere in which the spin vector currently resides, as illustrated in the bottom middle panel of Figure 2. Physically, this corresponds to setting the active surface as the adiabat for which the electronic wave function has the highest associated probability. As a result, MASH has purely deterministic dynamics, where the active surface changes whenever the spin vector crosses the equator. This guarantees that the MASH active surface is always consistent with the electronic wave function, thereby avoiding the inconsistency error of FSSH that is known to significantly degrade its accuracy.^5^

So far, we have considered only fully linearized mapping-based approaches,^31,49,50^ which contain a single set of electronic mapping variables and are generally able to describe the dynamics associated with the electronic populations. To correctly describe the dynamics of the coherences, partially linearized approaches are generally needed.^51,52^ These approaches use two sets of mapping variables, with each describing the electronic dynamics generated by the forward or backward propagator.^45,53,54^ The partially linearized version of spin-LSC is called spin-PLDM.^45,55^ Each set of spin-mapping variables in spin-PLDM is sampled independently from the same polar circles as in spin-LSC, such that the average of the two sets is distributed according to the top right panel of Figure 2. In particular, spin-PLDM trajectories can now be initialized with a force corresponding to the average of the two Born–Oppenheimer surfaces (pink region), which occurs whenever the two sets of spin-mapping variables are initialized on different polar circles. While a partially linearized version of MASH could be formulated in principle, this has yet to be developed.

To assess the ability of the different semiclassical mapping approaches to describe the nonadiabatic dynamics of an initial electronic coherence, we consider a typical photochemical scenario using the one-dimensional model system employed in refs (13) and (20). The Born–Oppenheimer surfaces and the initial coherent electronic wavepacket are depicted in panel 1 of Figure 1. The initial state has ∼80% of its weight on the lower adiabat, and its Gaussian profile corresponds to the ground vibrational eigenstate of the potential well located at *q* ≈ −2, all within the harmonic approximation. More details about the model are given in the Supporting Information.

We first consider the time evolution of the excited electronic-state population, which can be expressed as a single real-time correlation function of the form of eq 2, with . This means that the semiclassical mapping approaches can be used to calculate this quantity directly, with the results given in Figure 3. From the potentials shown in Figure 1, the wavepacket in the excited adiabatic state should oscillate about the coupling region at *q* = 0. As the parameters for this model are close to the Born–Oppenheimer limit, only a small amount of population is transferred at each crossing, giving rise to the step-like behavior of the exact population dynamics in Figure 3, which were computed using a split-operator approach.^56^

**Figure 3:**
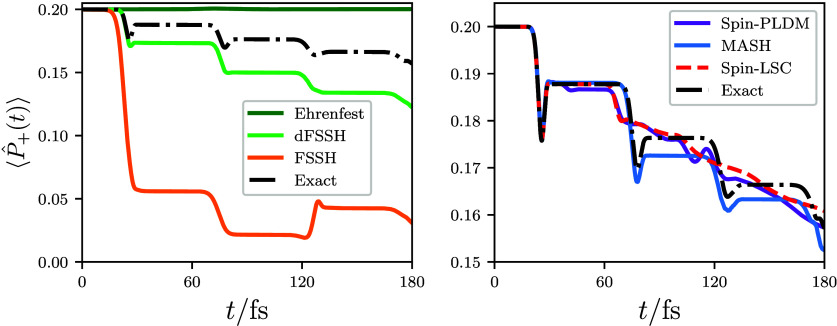
Population of the upper adiabatic electronic state as a function of time, calculated with a range of different independent-trajectory techniques. To compare with the most accurate FSSH algorithm, the associated electronic populations are always computed from the active surface and results using the energy-based decoherence correction^36,57^ with a decoherence parameter of 0.1 Hartree (dFSSH) are also included. Our Ehrenfest, FSSH, and dFSSH results match those presented in ref (13). We also encourage the reader to compare our results with the FSSH populations calculated using the electronic coefficients presented in ref (13).

In agreement with previous work,^13^ the left panel of Figure 3 shows that both Ehrenfest and FSSH are unable to describe the population dynamics originating from such an initial coherent electronic state. The composition of the initial state means that the major contribution to the Ehrenfest mean-field force comes from the ground-state surface so that those Ehrenfest trajectories that do reach the coupling region have relatively small nuclear velocities. As a result, the effective nonadiabatic coupling experienced by Ehrenfest trajectories at the coupling region is also small, explaining why Ehrenfest gives rise to negligible population transfer in this case. While FSSH does propagate the right fraction of trajectories on the excited-state surface, many of the trajectories nevertheless have an inconsistent electronic wave function (again due to the composition of the initial coherent state), which leads to the large error seen in the populations. Utilizing a decoherence correction (dFSSH) significantly reduces this error, but the corrected populations are still substantially different from the exact result.

As was shown in previous work on this model,^13^ one way to significantly improve the accuracy of the Ehrenfest and FSSH populations in this model is to initialize the system in an incoherent mixture of the two adiabatic states, which comes at the cost of neglecting a real-time description of the initial decoherence process. While such an approximate treatment may be suitable for systems giving rise to relatively fast decoherence, it is unlikely to be sufficient for systems exhibiting long-lived coherence or those driven by long laser pulses.

In contrast, all of the spin-mapping approaches closely match the exact result without needing to invoke approximate incoherent initial conditions. This improvement stems from the initial sampling introduced over the electronic spin-mapping variables. For the mean-field methods of spin-LSC and spin-PLDM, the initial sampling over the polar circles (Figure 2) leads to the correct fraction of trajectories experiencing the excited-state force. For MASH, the trajectories that propagate on the upper surface are those that are initialized with spin-mapping variables in the upper hemisphere, so that the electronic wave function is consistent with the nuclear propagation surface. The small differences in the electronic populations produced by the various spin-mapping methods can be better understood by considering the time-dependent nuclear density given in Figure 4. This quantity can also be expressed as a single-time correlation function with , where |*q*⟩ is an eigenstate of the nuclear position operator with eigenvalue *q* and  is the electronic identity operator. For the independent-trajectory approaches, the observable operator |*q*⟩⟨*q*| can be treated by histogramming the trajectories (see the Supporting Information for more details). Here we see that MASH captures the bifurication of the nuclear density on passing through the coupling region with essentially quantitative accuracy, while the mean-field methods in contrast lead to an incorrect “smearing out” of the density. One can thus also understand why MASH most accurately reproduces the step-like behavior of the exact populations at longer times in Figure 3 based on the underlying accuracy in the time evolution of the nuclear coordinate distribution. While dFSSH is also seen to reproduce the time-dependent nuclear density relatively well, we note that MASH achieves this without needing ad hoc decoherence corrections.

**Figure 4:**
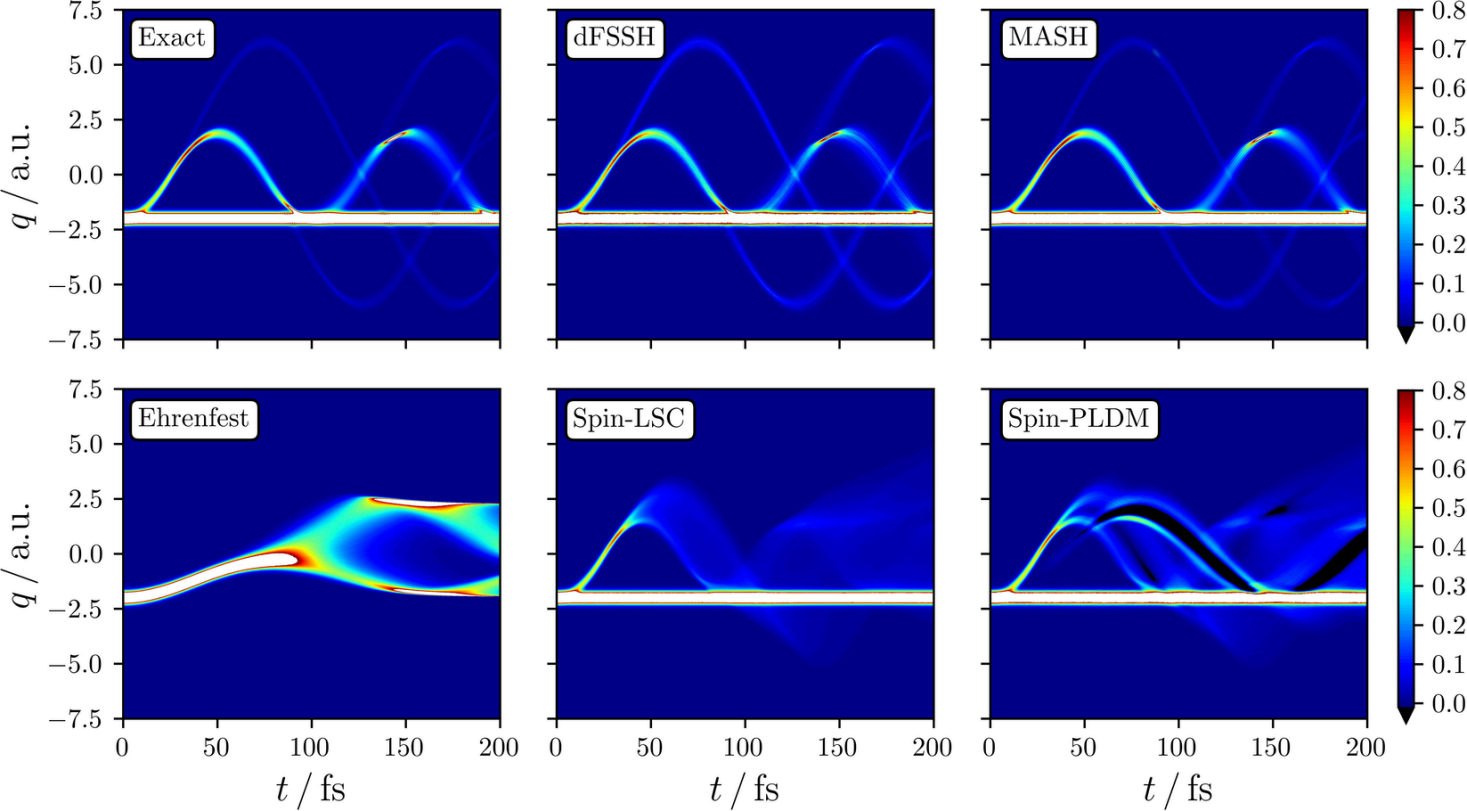
Time evolution of the total nuclear coordinate density, normalized so that *∫*d*q*_*t*_ ρ_nuc_(*q*_*t*_, *t*) = 1. Regions where the density exceeds a value of 0.8 are colored white, while those with an unphysical negative density are colored black.

We now consider the time evolution of the electronic coherences. One simple measure of the magnitude of the electronic coherence between two adiabatic states is obtained from the associated off-diagonal element of the electronic reduced density matrix, ρ_–+_(*t*). Unlike the electronic population and the nuclear density observables considered above, |ρ_–+_(*t*)| cannot be expressed as a single real-time correlation function. Therefore, to calculate this quantity with semiclassical mapping approaches, it must first be re-expressed in terms of correlation functions of the form of eq 2, which for a two-state system can be achieved as follows
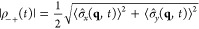
3where **σ̂**(**q**) is the Pauli spin matrices expressed in the adiabatic basis. Hence, any ensemble of independent trajectories should first be used to compute the correlation functions  and , and these quantities can subsequently be inserted into eq 3 to obtain the coherence measure.

**Figure 5:**
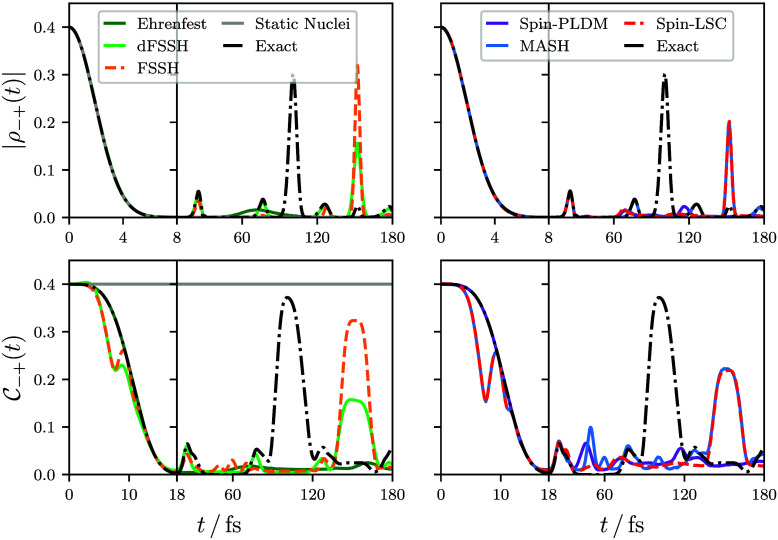
Two different electronic coherence measures, |ρ_–+_(*t*)| and , calculated with a range of different independent-trajectory techniques. The mathematical expressions for these measures are given by eqs 3 and 5, respectively. Our  measure is strongly related to that used in refs (13) and (58−60).

The top panels of Figure 5 give this coherence measure computed for the same model. These panels show that all of the independent-trajectory approaches can describe this initial decoherence behavior correctly. This is not so surprising, as the initial decay of |ρ_–+_(*t*)| is also captured in the limit of static nuclei and must therefore be dominated by pure dephasing.^61,62^ This is consistent with related findings for other model systems such as conjugated polymers.^63^

While |ρ_–+_(*t*)| is useful due to its close connection with linear spectroscopic signatures,^52^ it nevertheless does not offer the most rigorous definition of electronic coherence. To see this, |ρ_–+_(*t*)| can be expressed in terms of the time-dependent wave function coefficients, *c*_*j*_(**q**_*t*_, *t*), as
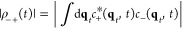
4which are themselves defined by the expression for the wave function appearing in eq 1. Because the coefficients are not positive functions of **q**_*t*_, |ρ_–+_(*t*)| can in principle be zero even if the coefficients on different surfaces are spatially overlapping. To resolve this issue, the order of the integral and the magnitude in eq 4 can be interchanged to give^13,58−60^
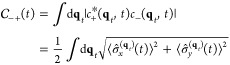
5where  is a product of a Pauli spin matrix and a projector onto the specific eigenstate of the nuclear position operator, |**q**⟩. Another advantage of the  measure is that the pure dephasing contribution to the initial decoherence is completely removed, so that decoherence effects arising from the nuclear motion can be more easily investigated.

We proceed by considering the initial dynamics of this coherence measure, shown in the bottom panels of Figure 5. All of the independent-trajectory approaches give some initial decay of the  measure, demonstrating that they all capture aspects of decoherence beyond the pure dephasing limit. This may seem surprising given that individual trajectories are known to remain “overcoherent” after a nonadiabatic transition.^64−66^ Indeed, if eq 5 was calculated using just a single trajectory,  would be essentially constant as a function of time and no decoherence would be observed. Decoherence therefore arises as a result of ensemble averaging. While the contribution to  and  from each trajectory is in general non-zero, the sign can vary, leading to phase cancellation^61^ among trajectories such that the ensemble-averaged values can be zero. This also introduces effects beyond pure dephasing as phase cancellation can also occur between trajectories that begin with different nuclear configurations but end up with the same configuration at some later time, *t*. This highlights an important philosophical point underpinning most trajectory-based approaches: physical meaning can only be ascribed to average quantities derived from the trajectory ensemble, and not to the individual trajectories themselves.^67^ While individual trajectories do remain unphysically overcoherent after a nonadiabatic transition, Figure 5 highlights that the trajectory ensemble displays the correct decoherence behavior for a wide range of methods. This is also true for the FSSH results, for which no decoherence correction was applied.^b^

However, not all of the independent-trajectory approaches can exactly describe the decay of this measure. The approaches that can (i.e., Ehrenfest and spin-PLDM) are those that initially propagate at least some of their trajectories on the average surface. This highlights one of the major advantages of partially linearized approaches, like spin-PLDM, which can simultaneously describe the population and coherence dynamics relatively accurately by initially propagating trajectories on both single and average Born–Oppenheimer surfaces.

For all of the independent-trajectory approaches, the ability to describe the longer-time coherence dynamics for both measures is mixed. We first consider the smaller transient coherence peaks, which originate in the exact dynamics from nonadiabatic transitions (see panel 3 of Figure 1). The first such peak is well captured by all of the mapping-based approaches, and subsequent peaks are almost perfectly captured by dFSSH and MASH.

Notably, none of the methods can reproduce the large recoherence peak at ≈100 fs, which arises from the re-overlap of the excited-state wavepacket with the stationary ground-state wavepacket at *q* ≈ −2. Additionally, most of the independent-trajectory approaches show several spurious recoherence peaks in the  measure, which have no analogue in the exact dynamics. To better understand the source of these discrepancies, we also consider the coherence dynamics in the Born–Oppenheimer limit, with the results given in Figure S1. Note that the recoherence behavior of all of the independent-trajectory methods in the Born–Oppenheimer limit largely matches their behavior in the full system. In particular, spin-PLDM reduces to the so-called Wigner-averaged classical limit (WACL)^30−32^ in the Born–Oppenheimer limit.^52^ Given that WACL only differs from the exact Born–Oppenheimer dynamics as a result of its classical-nuclear approximation, this suggests that the classical-nuclear approximation is the main reason behind why the recoherences are difficult to describe with independent trajectories.

Finally, to further establish the relevance of these findings for the coherent photoexcited dynamics of a real molecule, we consider the bis(methylene) adamantyl cation (BMA) using a two-dimensional linear vibronic model that includes a conical intersection.^69^ The dynamics is initialized in an electronic coherence, exactly as in the previously considered model, and the time-dependent excited-state populations are shown in Figure 6. The BMA model gives rise to relatively weak diabatic coupling, in contrast to the relatively weak nonadiabatic coupling of the first model, meaning that the dynamics exhibited by the two systems are quantitatively quite different. Nevertheless, there are still clear qualitative similarities in how well the different methods describe the dynamics proceeding from an initial electronic coherence. As was observed for the one-dimensional model considered previously, both Ehrenfest and FSSH exhibit large errors in the dynamics originating from the coherent initial conditions, while all of the spin-mapping approaches offer significant improvements over the original methods, giving results in close agreement with the exact population dynamics. Interestingly, applying a decoherence correction offers almost no improvement to the FSSH result in this case, illustrating that such corrections cannot always be relied upon to fix the inconsistency error in the FSSH results.

**Figure 6:**
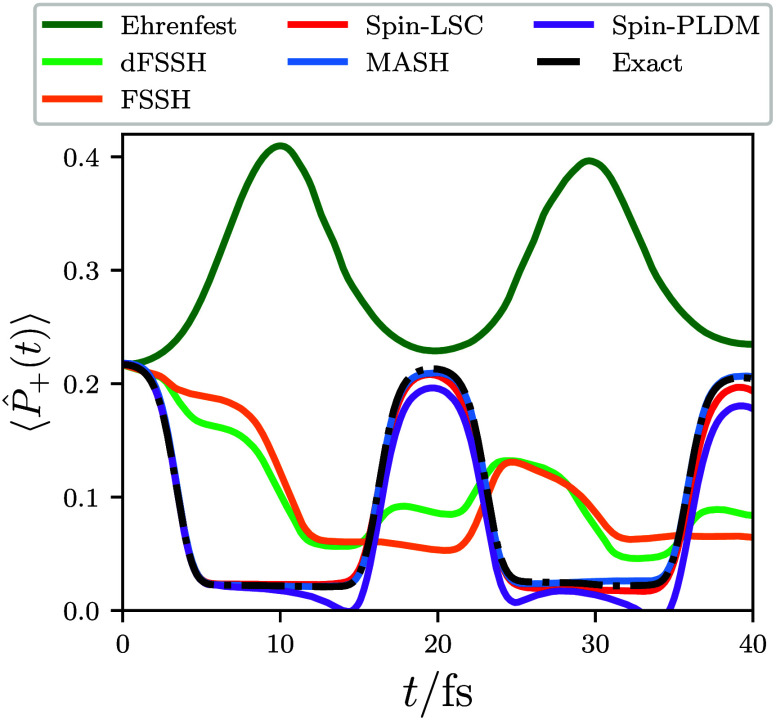
Time evolution of the population of the upper adiabatic state in the bis(methylene) adamantyl cation (BMA), calculated using a two-dimensional linear vibronic coupling model containing a conical intersection.

In conclusion, we have assessed the ability of a range of independent-trajectory nonadiabatic dynamics approaches to describe the dynamics proceeding from a coherent electronic state using models relevant for photochemical systems. The ability to treat these initial conditions provides a first step toward a more accurate description of the effects of tailored electromagnetic pulses within nonadiabatic simulations, which is a crucial step for providing a more direct connection with cutting-edge experiments.

To correctly describe an initial electronic coherence with independent-trajectory approaches, we have found that it is important to introduce an initial sampling over the electronic phase space, as is naturally incorporated within the semiclassical mapping framework. In particular, we considered all possible flavors of the spin-mapping approach, all of which were found to lead to the right fraction of trajectories being initialized on each adiabatic surface, as required to reproduce the correct population dynamics. MASH was particularly successful for this model, where the surface-hopping force meant that the approach could accurately describe the nonadiabatic transitions associated with multiple crossings of the localized coupling region. However, all of the spin-mapping methods were seen to be a significant upgrade on the more commonly used Ehrenfest, FSSH, and dFSSH approaches, which in contrast failed to correctly reproduce these aspects of the dynamics. All of the spin-mapping approaches considered here have multistate extensions, so similar improvements in accuracy would be expected from these methods in systems containing more than two electronic states. While we have exclusively focused on the spin-mapping formalism here, our conclusions would have been identical for the majority of other mapping representations.

We also showed that independent-trajectory approaches can reproduce the correct decoherence behavior. Even though the individual trajectories of these approaches remain overcoherent, decoherence is nevertheless included via phase cancellation between them through ensemble averaging in the construction of the relevant correlation functions. However, not every independent-trajectory approach was able to perfectly describe all aspects of the coherence measures. In particular, for the more stringent  measure, it was necessary to propagate at least some trajectories on the average Born–Oppenheimer surface to completely capture the initial decay. This illustrates one of the advantages of partially linearized approaches over their fully linearized counterparts.^51^ Similar findings were also found in previous work when calculating real-time dipole–dipole correlation functions for optical spectra, where partially linearized approaches also offered a significant advantage.^52^ Given the advantages of describing the electronic population dynamics in photochemical simulations with MASH over analogous mean-field approaches, it is expected that similar advantages would also arise from a partially linearized version of MASH for computing optical spectra in such systems. Obtaining such a method will be one aspect of future work.

Another aspect of future work will be to develop mapping-based approaches that can couple an explicit electromagnetic pulse. For mean-field approaches, this extension is straightforward and has already been achieved for fully and partially linearized mapping approaches such as PBME and FBTS,^70^ as well as spin-LSC.^71^ We are currently working on the necessary developments of the algorithm to include this effect correctly for surface-hopping methods like MASH.

Finally, the classical-nuclear approximation is observed to lead to the absence of recoherence phenomena in the dynamics of independent-trajectory approaches. In the majority of realistic (high-dimensional) systems, recoherence phenomena are suppressed, and one may expect that this deficiency will not be a major problem. However, the classical-nuclear approximation is also known to “wash out” other coherence-related phenomena, such as contributions to dipole–dipole correlation functions that give rise to vibronic progressions in optical spectra.^72−74^ For this application, it would therefore be useful to develop extensions of nonequilibrium trajectory-based approaches that go beyond the classical-nuclear approximation. While a number of ring-polymer extensions to nonadiabatic trajectory-based approaches have been developed for simulating equilibrium dynamics,^75−81^ to the best of our knowledge there are currently no established methods for tackling the nonequilibrium regime.

## Supporting Information Available

All of the necessary information to reproduce the results in the main text (PDF)

## Footnotes

a In the following, we refer to the diagonal elements of the density matrix as populations and the off-diagonal elements as coherences.

b However, we also clarify that this does not mean that decoherence corrections are never needed in FSSH. There are well-known cases in which decoherence corrections are required to even ensure the trajectory ensemble is physically correct.^68^

## References

[ref1] Shapiro M.; Brumer P. Quantum control of molecular processes; John Wiley & Sons, 2012.

[ref2] McLachlan A. D. A variational solution of the time-dependent Schrodinger equation. Mol. Phys. 1964, 8, 39–44. 10.1080/00268976400100041.

[ref3] Grunwald R.; Kelly A.; Kapral R. Energy Transfer Dynamics in Biomaterial Systems; Springer: Berlin, 2009; pp 383–413.

[ref4] Tully J. C. Molecular dynamics with electronic transitions. J. Chem. Phys. 1990, 93, 1061–1071. 10.1063/1.459170.

[ref5] Subotnik J. E.; Jain A.; Landry B.; Petit A.; Ouyang W.; Bellonzi N. Understanding the surface hopping view of electronic transitions and decoherence. Annu. Rev. Phys. Chem. 2016, 67, 387–417. 10.1146/annurev-physchem-040215-112245. 27215818

[ref6] Fiedlschuster T.; Handt J.; Gross E. K. U.; Schmidt R. Surface hopping in laser-driven molecular dynamics. Phys. Rev. A 2017, 95, 063424 10.1103/PhysRevA.95.063424.

[ref7] Mignolet B.; Curchod B. F. E. Excited-State Molecular Dynamics Triggered by Light Pulses—Ab Initio Multiple Spawning vs Trajectory Surface Hopping. J. Phys. Chem. A 2019, 123, 3582–3591. 10.1021/acs.jpca.9b00940. 30938525

[ref8] Suchan J.; Hollas D.; Curchod B. F. E.; Slavíček P. On the importance of initial conditions for excited-state dynamics. Faraday Discuss. 2018, 212, 307–330. 10.1039/C8FD00088C. 30259011

[ref9] Barbatti M. Simulation of Excitation by Sunlight in Mixed Quantum-Classical Dynamics. J. Chem. Theory Comput. 2020, 16, 4849–4856. 10.1021/acs.jctc.0c00501. 32579345 PMC7426902

[ref10] Messiah Quantum Mechanics; North-Holland: Amsterdam, 1961.

[ref11] Mignolet B.; Curchod B. F. E. A walk through the approximations of ab initio multiple spawning. J. Chem. Phys. 2018, 148, 134110 10.1063/1.5022877. 29626896

[ref12] Tran T.; Ferté A.; Vacher M. Simulating Attochemistry: Which Dynamics Method to Use?. J. Phys. Chem. Lett. 2024, 15, 3646–3652. 10.1021/acs.jpclett.4c00106. 38530933 PMC11000647

[ref13] Villaseco Arribas E.; Maitra N. T.; Agostini F. Nonadiabatic dynamics with classical trajectories: The problem of an initial coherent superposition of electronic states. J. Chem. Phys. 2024, 160, 054102 10.1063/5.0186984. 38310471

[ref14] Ben-Nun M.; Quenneville J.; Martínez T. J. Ab Initio Multiple Spawning: Photochemistry from First Principles Quantum Molecular Dynamics. J. Phys. Chem. A 2000, 104, 5161–5175. 10.1021/jp994174i.

[ref15] Richings G. W.; Polyak I.; Spinlove K. E.; Worth G. A.; Burghardt I.; Lasorne B. Quantum dynamics simulations using Gaussian wavepackets: the vMCG method. Int. Rev. Phys. Chem. 2015, 34, 269–308. 10.1080/0144235X.2015.1051354.

[ref16] Makhov D. V.; Symonds C.; Fernandez-Alberti S.; Shalashilin D. V. Ab initio quantum direct dynamics simulations of ultrafast photochemistry with Multiconfigurational Ehrenfest approach. Chem. Phys. 2017, 493, 200–218. 10.1016/j.chemphys.2017.04.003.

[ref17] Curchod B. F. E.; Martínez T. J. Ab Initio Nonadiabatic Quantum Molecular Dynamics. Chem. Rev. 2018, 118, 3305–3336. 10.1021/acs.chemrev.7b00423. 29465231

[ref18] Donoso A.; Martens C. C. Simulation of Coherent Nonadiabatic Dynamics Using Classical Trajectories. J. Phys. Chem. A 1998, 102, 4291–4300. 10.1021/jp980219o.

[ref19] Abedi A.; Agostini F.; Gross E. K. U. Mixed quantum-classical dynamics from the exact decomposition of electron-nuclear motion. Europhys. Lett. 2014, 106, 33001 10.1209/0295-5075/106/33001.

[ref20] Pieroni C.; Agostini F. Nonadiabatic Dynamics with Coupled Trajectories. J. Chem. Theory Comput. 2021, 17, 5969–5991. 10.1021/acs.jctc.1c00438. 34506154

[ref21] Dupuy L.; Rikus A.; Maitra N. T. Exact-Factorization-Based Surface Hopping without Velocity Adjustment. J. Phys. Chem. Lett. 2024, 15, 2643–2649. 10.1021/acs.jpclett.4c00115. 38422391

[ref22] Abedi A.; Maitra N. T.; Gross E. K. U. Exact Factorization of the Time-Dependent Electron-Nuclear Wave Function. Phys. Rev. Lett. 2010, 105, 123002 10.1103/PhysRevLett.105.123002. 20867633

[ref23] Miller W. H.; McCurdy C. W. Classical trajectory model for electronically nonadiabatic collision phenomena. A classical analog for electronic degrees of freedom. J. Chem. Phys. 1978, 69, 5163–5173. 10.1063/1.436463.

[ref24] Stock G.; Thoss M. Semiclassical description of nonadiabatic quantum dynamics. Phys. Rev. Lett. 1997, 78, 578–581. 10.1103/PhysRevLett.78.578. 11580635

[ref25] Stock G.; Thoss M. Classical description of nonadiabatic quantum dynamics. Adv. Chem. Phys. 2005, 131, 243–376. 10.1002/0471739464.ch5.

[ref26] Kim H.; Nassimi A.; Kapral R. Quantum-classical Liouville dynamics in the mapping basis. J. Chem. Phys. 2008, 129, 084102 10.1063/1.2971041. 19044813

[ref27] Kelly A.; van Zon R.; Schofield J.; Kapral R. Mapping quantum-classical Liouville equation: Projectors and trajectories. J. Chem. Phys. 2012, 136, 084101 10.1063/1.3685420. 22380026

[ref28] Runeson J. E.; Richardson J. O. Spin-mapping approach for nonadiabatic molecular dynamics. J. Chem. Phys. 2019, 151, 044119 10.1063/1.5100506. 31370524

[ref29] Kapral R.; Ciccotti G. Mixed quantum-classical dynamics. J. Chem. Phys. 1999, 110, 8919–8929. 10.1063/1.478811.

[ref30] Egorov S. A.; Rabani E.; Berne B. J. Nonradiative relaxation processes in condensed phases: Quantum versus classical baths. J. Chem. Phys. 1999, 110, 5238–5248. 10.1063/1.478420.

[ref31] Shi Q.; Geva E. Nonradiative electronic relaxation rate constants from approximations based on linearizing the path-integral forward-backward action. J. Phys. Chem. A 2004, 108, 6109–6116. 10.1021/jp049547g.

[ref32] Shi Q.; Geva E. A comparison between different semiclassical approximations for optical response functions in nonpolar liquid solutions. J. Chem. Phys. 2005, 122, 064506 10.1063/1.1843813. 15740387

[ref33] Hammes-Schiffer S.; Tully J. C. Proton transfer in solution: Molecular dynamics with quantum transitions. J. Chem. Phys. 1994, 101, 4657–4667. 10.1063/1.467455.

[ref34] Bittner E. R.; Rossky P. J. Quantum decoherence in mixed quantum-classical systems: Nonadiabatic processes. J. Chem. Phys. 1995, 103, 8130–8143. 10.1063/1.470177.

[ref35] Jasper A. W.; Truhlar D. G. Electronic decoherence time for non-Born-Oppenheimer trajectories. J. Chem. Phys. 2005, 123, 064103 10.1063/1.1995695. 16122296

[ref36] Granucci G.; Persico M.; Zoccante A. Including quantum decoherence in surface hopping. J. Chem. Phys. 2010, 133, 134111 10.1063/1.3489004. 20942527

[ref37] Subotnik J. E.; Shenvi N. A new approach to decoherence and momentum rescaling in the surface hopping algorithm. J. Chem. Phys. 2011, 134, 024105 10.1063/1.3506779. 21241078

[ref38] Shenvi N.; Subotnik J. E.; Yang W. Simultaneous-trajectory surface hopping: A parameter-free algorithm for implementing decoherence in nonadiabatic dynamics. J. Chem. Phys. 2011, 134, 144102 10.1063/1.3575588. 21495737

[ref39] Subotnik J. E. Fewest-Switches Surface Hopping and Decoherence in Multiple Dimensions. J. Phys. Chem. A 2011, 115, 12083–12096. 10.1021/jp206557h. 21995423

[ref40] Jaeger H. M.; Fischer S.; Prezhdo O. V. Decoherence-induced surface hopping. J. Chem. Phys. 2012, 137, 22A545 10.1063/1.4757100. 23249082

[ref41] Vindel-Zandbergen P.; Ibele L. M.; Ha J.-K.; Min S. K.; Curchod B. F. E.; Maitra N. T. Study of the Decoherence Correction Derived from the Exact Factorization Approach for Nonadiabatic Dynamics. J. Chem. Theory Comput. 2021, 17, 3852–3862. 10.1021/acs.jctc.1c00346. 34138553 PMC8280698

[ref42] Meyer H.-D.; Miller W. H. Classical models for electronic degrees of freedom: Derivation via spin analogy and application to F*+H_2_ → F+H_2_. J. Chem. Phys. 1979, 71, 2156–2169. 10.1063/1.438598.

[ref43] He X.; Liu J. A new perspective for nonadiabatic dynamics with phase space mapping models. J. Chem. Phys. 2019, 151, 024105 10.1063/1.5108736. 31301706

[ref44] Runeson J. E.; Richardson J. O. Generalized spin mapping for quantum-classical dynamics. J. Chem. Phys. 2020, 152, 084110 10.1063/1.5143412. 32113368

[ref45] Mannouch J. R.; Richardson J. O. A partially linearized spin-mapping approach for nonadiabatic dynamics. I. Derivation of the theory. J. Chem. Phys. 2020, 153, 194109 10.1063/5.0031168. 33218231

[ref46] Mannouch J. R.; Richardson J. O. A mapping approach to surface hopping. J. Chem. Phys. 2023, 158, 104111 10.1063/5.0139734. 36922129

[ref47] Lawrence J. E.; Mannouch J. R.; Richardson J. O. A size-consistent multi-state mapping approach to surface hopping. J. Chem. Phys. 2024, 160, 244112 10.1063/5.0208575. 38940540

[ref48] Runeson J. E.; Manolopoulos D. E. A multi-state mapping approach to surface hopping. J. Chem. Phys. 2023, 159, 094115 10.1063/5.0158147. 37675848

[ref49] Sun X.; Wang H.; Miller W. H. Semiclassical theory of electronically nonadiabatic dynamics: Results of a linearized approximation to the initial value representation. J. Chem. Phys. 1998, 109, 7064–7074. 10.1063/1.477389.

[ref50] Miller W. H. The Semiclassical Initial Value Representation: A Potentially Practical Way for Adding Quantum Effects to Classical Molecular Dynamics Simulations. J. Phys. Chem. A 2001, 105, 2942–2955. 10.1021/jp003712k.

[ref51] Miller W. H. Perspective: Quantum or classical coherence?. J. Chem. Phys. 2012, 136, 210901 10.1063/1.4727849. 22697519

[ref52] Mannouch J. R.; Richardson J. O. A partially linearized spin-mapping approach for simulating nonlinear optical spectra. J. Chem. Phys. 2022, 156, 024108 10.1063/5.0077744. 35032975

[ref53] Hsieh C.-Y.; Kapral R. Nonadiabatic dynamics in open quantum-classical systems: Forward-backward trajectory solution. J. Chem. Phys. 2012, 137, 22A507 10.1063/1.4736841. 23249044

[ref54] Huo P.; Coker D. F. Consistent schemes for non-adiabatic dynamics derived from partial linearized density matrix propagation. J. Chem. Phys. 2012, 137, 22A535 10.1063/1.4748316. 23249072

[ref55] Mannouch J. R.; Richardson J. O. A partially linearized spin-mapping approach for nonadiabatic dynamics. II. Analysis and comparison with related approaches. J. Chem. Phys. 2020, 153, 194110 10.1063/5.0031173. 33218246

[ref56] Tannor D. J. Introduction to Quantum Mechanics: A Time-Dependent Perspective; University Science Books: Sausalito, CA, 2007.

[ref57] Granucci G.; Persico M. Critical appraisal of the fewest switches algorithm for surface hopping. J. Chem. Phys. 2007, 126, 134114 10.1063/1.2715585. 17430023

[ref58] Vacher M.; Bearpark M. J.; Robb M. A.; Malhado J. a. P. Electron Dynamics upon Ionization of Polyatomic Molecules: Coupling to Quantum Nuclear Motion and Decoherence. Phys. Rev. Lett. 2017, 118, 083001 10.1103/PhysRevLett.118.083001. 28282194

[ref59] Curchod B. F. E.; Agostini F.; Tavernelli I. CT-MQC – a coupled-trajectory mixed quantum/classical method including nonadiabatic quantum coherence effects. Eur. Phys. J. B 2018, 91, 168 10.1140/epjb/e2018-90149-x.

[ref60] Arribas E. V.; Maitra N. T. Electronic Coherences in Molecules: The Projected Nuclear Quantum Momentum as a Hidden Agent. arXiv 2024, 10.48550/arXiv.2405.00649. 39714655

[ref61] Fiete G. A.; Heller E. J. Semiclassical theory of coherence and decoherence. Phys. Rev. A 2003, 68, 022112 10.1103/PhysRevA.68.022112.

[ref62] Vacher M.; Steinberg L.; Jenkins A. J.; Bearpark M. J.; Robb M. A. Electron dynamics following photoionization: Decoherence due to the nuclear-wave-packet width. Phys. Rev. A 2015, 92, 040502 10.1103/PhysRevA.92.040502.

[ref63] Hu W.; Gu B.; Franco I. Lessons on electronic decoherence in molecules from exact modeling. J. Chem. Phys. 2018, 148, 134304 10.1063/1.5004578. 29626859

[ref64] Bittner E. R.; Rossky P. J. Quantum decoherence in mixed quantum-classical systems: Nonadiabatic processes. J. Chem. Phys. 1995, 103, 8130–8143. 10.1063/1.470177.

[ref65] Fang J.-Y.; Hammes-Schiffer S. Improvement of the Internal Consistency in Trajectory Surface Hopping. J. Phys. Chem. A 1999, 103, 9399–9407. 10.1021/jp991602b.

[ref66] Subotnik J. E.; Shenvi N. Decoherence and surface hopping: When can averaging over initial conditions help capture the effects of wave packet separation?. J. Chem. Phys. 2011, 134, 244114 10.1063/1.3603448. 21721619

[ref67] Persico M.; Granucci G. An overview of nonadiabatic dynamics simulations methods, with focus on the direct approach versus the fitting of potential energy surfaces. Theor. Chem. Acc. 2014, 133, 1526 10.1007/s00214-014-1526-1.

[ref68] Lawrence J. E.; Mannouch J. R.; Richardson J. O. Recovering Marcus Theory Rates and Beyond without the Need for Decoherence Corrections: The Mapping Approach to Surface Hopping. J. Phys. Chem. Lett. 2024, 15, 707–716. 10.1021/acs.jpclett.3c03197. 38214476 PMC10823533

[ref69] Ryabinkin I. G.; Joubert-Doriol L.; Izmaylov A. F. When do we need to account for the geometric phase in excited state dynamics?. J. Chem. Phys. 2014, 140, 214116 10.1063/1.4881147. 24907999

[ref70] Martinez F.; Rekik N.; Hanna G. Simulation of nonlinear optical signals via approximate solutions of the quantum–classical Liouville equation: Application to the pump–probe spectroscopy of a condensed phase electron transfer reaction. Chem. Phys. Lett. 2013, 573, 77–83. 10.1016/j.cplett.2013.04.018.

[ref71] Runeson J. E.; Mannouch J. R.; Amati G.; Fiechter M. R.; Richardson J. O. Spin-mapping methods for simulating ultrafast nonadiabatic dynamics. Chimia 2022, 76, 582–588. 10.2533/chimia.2022.582. 38069729

[ref72] McRobbie P. L.; Geva E. A benchmark study of different methods for calculating one-and two-dimensional optical spectra. J. Phys. Chem. A 2009, 113, 10425–10434. 10.1021/jp905305t. 19775171

[ref73] Karsten S.; Ivanov S. D.; Bokarev S. I.; Kühn O. Quasi-classical approaches to vibronic spectra revisited. J. Chem. Phys. 2018, 148, 102337 10.1063/1.5011764. 29544262

[ref74] Lively K.; Albareda G.; Sato S. A.; Kelly A.; Rubio A. Simulating vibronic spectra without born–oppenheimer surfaces. J. Phys. Chem. Lett. 2021, 12, 3074–3081. 10.1021/acs.jpclett.1c00073. 33750137 PMC8020382

[ref75] Shushkov P.; Li R.; Tully J. C. Ring polymer molecular dynamics with surface hopping. J. Chem. Phys. 2012, 137, 22A549 10.1063/1.4766449. 23249086

[ref76] Richardson J. O.; Thoss M. Communication: Nonadiabatic ring-polymer molecular dynamics. J. Chem. Phys. 2013, 139, 031102 10.1063/1.4816124. 23883003

[ref77] Ananth N. Mapping variable ring polymer molecular dynamics: A path-integral based method for nonadiabatic processes. J. Chem. Phys. 2013, 139, 124102 10.1063/1.4821590. 24089745

[ref78] Richardson J. O.; Meyer P.; Pleinert M.-O.; Thoss M. An analysis of nonadiabatic ring-polymer molecular dynamics and its application to vibronic spectra. Chem. Phys. 2017, 482, 124–134. 10.1016/j.chemphys.2016.09.036.

[ref79] Chowdhury S. N.; Huo P. Coherent State Mapping Ring-Polymer Molecular Dynamics for Non-Adiabatic quantum propagations. J. Chem. Phys. 2017, 147, 214109 10.1063/1.4995616. 29221374

[ref80] Bossion D.; Chowdhury S. N.; Huo P. Non-adiabatic ring polymer molecular dynamics with spin mapping variables. J. Chem. Phys. 2021, 154, 184106 10.1063/5.0051456. 34241014

[ref81] Bossion D.; Chowdhury S. N.; Huo P. Non-adiabatic ring polymer molecular dynamics in the phase space of the SU (N) Lie group. J. Chem. Phys. 2023, 158, 044123 10.1063/5.0133970. 36725494

