# Supplementary material for: Toward a Correct Description of Initial Electronic Coherence in Nonadiabatic Dynamics Simulations
Source: J Phys Chem Lett. 2024 Nov 14;15(46):11687–95. doi: 10.1021/acs.jpclett.4c02418 (PMC11587105; doi:10.1021/acs.jpclett.4c02418)
Supplement: Supplementary file 1 — jz4c02418_si_001.pdf [file jz4c02418_si_001.pdf]

Supporting Information: Towards a correct description of initial electronic coherence in nonadiabatic dynamics simulations

Jonathan R. Mannouch* and Aaron Kelly*

Hamburg Center for Ultrafast Imaging, Universität Hamburg and the Max Planck Institute for the Structure and Dynamics of Matter, Luruper Chaussee 149, 22761 Hamburg, Germany

E-mail: jonathan.mannouch@mpsd.mpg.de; aaron.kelly@mpsd.mpg.de

Hamiltonian and Initial Density Matrix

For any system consisting of two diabatic electronic states coupled to a set of nuclear degrees of freedom, the associated Hamiltonian can be expressed in atomic units as

$$\hat{H} = \sum_{j=1}^{N} \frac{p_j^2}{2m} + \bar{V}(\boldsymbol{q})\hat{\mathcal{I}} + \Delta(\boldsymbol{q})\hat{\sigma}_x^{\text{diab}} + \kappa(\boldsymbol{q})\hat{\sigma}_z^{\text{diab}}, \tag{S1}$$

where $\boldsymbol{q}$ and $\boldsymbol{p}$ are the nuclear positions and momenta of dimension N, expressed in mass-weighted coordinates so that each degree of freedom has the same mass, m. In addition, $\hat{\boldsymbol{\sigma}}^{\text{diab}}$ are the standard Pauli spin matrices expressed in the diabatic basis

$$\hat{\sigma}_x^{\text{diab}} = |\psi_1\rangle\langle\psi_2| + |\psi_2\rangle\langle\psi_1|, \tag{S2a}$$

$$\hat{\sigma}_y^{\text{diab}} = i(|\psi_1\rangle\langle\psi_2| - |\psi_2\rangle\langle\psi_1|), \tag{S2b}$$

$$\hat{\sigma}_z^{\text{diab}} = |\psi_2\rangle\langle\psi_2| - |\psi_1\rangle\langle\psi_1|, \tag{S2c}$$

which along with the identity operator, $\hat{\mathcal{I}}$, form a complete set of Hermitian operators for two-state systems. Finally, the sign of $\kappa(\boldsymbol{q})$ is chosen so that $\kappa(\bar{\boldsymbol{q}}) > 0$, where $\bar{\boldsymbol{q}}$ is the average nuclear

position of the initial quantum state. This means that $|\psi_2\rangle$ is the highest-energy diabat at this nuclear geometry.

The adiabatic states associated with this Hamiltonian are given by

$$|\psi_+(\boldsymbol{q})\rangle = \mathrm{e}^{i\theta(\boldsymbol{q})/2}\left(\cos\left(\theta(\boldsymbol{q})/2\right)|\psi_2\rangle + \sin\left(\theta(\boldsymbol{q})/2\right)|\psi_1\rangle\right), \tag{S3a}$$

$$|\psi_-(\boldsymbol{q})\rangle = \mathrm{e}^{i\theta(\boldsymbol{q})/2}\left(-\sin\left(\theta(\boldsymbol{q})/2\right)|\psi_2\rangle + \cos\left(\theta(\boldsymbol{q})/2\right)|\psi_1\rangle\right), \tag{S3b}$$

where $\tan\theta(\boldsymbol{q}) = \Delta(\boldsymbol{q})/\kappa(\boldsymbol{q})$ with $-\pi < \theta(\boldsymbol{q}) \leq \pi$ and the + and − indices refer to the upper and lower states. From these expressions, the adiabatic potential energies, $V_\pm(\boldsymbol{q})$, and the nonadiabatic coupling vector (NACV), $d_j(\boldsymbol{q})$, can be expressed as

$$V_z(\boldsymbol{q}) = \sqrt{\Delta(\boldsymbol{q})^2 + \kappa(\boldsymbol{q})^2}, \tag{S4a}$$

$$d_j(\boldsymbol{q}) = \left\langle \psi_+(\boldsymbol{q}) \,\middle|\, \frac{\partial\psi_-(\boldsymbol{q})}{\partial q_j} \right\rangle = \frac{\Delta(\boldsymbol{q})\frac{\partial\kappa(\boldsymbol{q})}{\partial q_j} - \kappa(\boldsymbol{q})\frac{\partial\Delta(\boldsymbol{q})}{\partial q_j}}{2(\Delta(\boldsymbol{q})^2 + \kappa(\boldsymbol{q})^2)}, \tag{S4b}$$

where $V_\pm(\boldsymbol{q}) = \bar{V}(\boldsymbol{q}) \pm V_z(\boldsymbol{q})$.

As in Ref. S1, the dynamics is initialized in a product state corresponding to the electronic wavefunction, $|\psi\rangle = \sqrt{\frac{1}{2}(1-\Delta P)}\,|\psi_2\rangle + \sqrt{\frac{1}{2}(1+\Delta P)}\,|\psi_1\rangle$, and the nuclear ground state of the diabatic potential, $V_1(\boldsymbol{q}) = \bar{V}(\boldsymbol{q}) - \kappa(\boldsymbol{q})$. The partial Wigner transform of the initial density matrix is then

$$\hat{\rho}^{\mathrm{W}}(\boldsymbol{q},\boldsymbol{p}) = \hat{\rho}_{\mathrm{el}}\,\rho_{\mathrm{nuc}}(\boldsymbol{q},\boldsymbol{p}), \tag{S5a}$$

$$\hat{\rho}_{\mathrm{el}} = |\psi\rangle\langle\psi| = \tfrac{1}{2}\left[\hat{\mathcal{I}} - \Delta P\hat{\sigma}_z^{\mathrm{diab}} + \sqrt{1-\Delta P^2}\,\hat{\sigma}_x^{\mathrm{diab}}\right], \tag{S5b}$$

$$\rho_{\mathrm{nuc}}(\boldsymbol{q},\boldsymbol{p}) = \prod_{j=1}^{N}\frac{1}{\pi}\mathrm{e}^{-\frac{k_j(q_j-\bar{q}_j)^2}{\omega_j}}\,\mathrm{e}^{-\frac{p_j^2}{m\omega_j}}, \tag{S5c}$$

where $\Delta P = 0.6$ is the electronic population difference between the lower and upper diabatic states and $\omega_j = \sqrt{k_j/m}$ is the harmonic frequency associated with mode j.

The initial electronic density matrix, $\hat{\rho}_{\text{el}}$, can also be expressed in the adiabatic basis

$$\hat{\rho}_{\text{el}} = \tfrac{1}{2}\left[\hat{\mathcal{I}} + A_z(\boldsymbol{q})\hat{\sigma}_z(\boldsymbol{q}) + A_x(\boldsymbol{q})\hat{\sigma}_x(\boldsymbol{q})\right], \tag{S6a}$$

$$A_x(\boldsymbol{q}) = \frac{(\kappa(\boldsymbol{q})\sqrt{1-\Delta P^2} + \Delta(\boldsymbol{q})\Delta P)}{V_z(\boldsymbol{q})}, \tag{S6b}$$

$$A_z(\boldsymbol{q}) = \frac{(\Delta(\boldsymbol{q})\sqrt{1-\Delta P^2} - \kappa(\boldsymbol{q})\Delta P)}{V_z(\boldsymbol{q})}. \tag{S6c}$$

where $\hat{\boldsymbol{\sigma}}(\boldsymbol{q})$ are the Pauli spin matrices defined in the adiabatic basis, which are related to $\hat{\boldsymbol{\sigma}}^{\text{diab}}$ by a unitary transformation.[S2]

Quasiclassical Trajectory Techniques

In this section, we define the equations of motion and observables associated with all of the quasiclassical-trajectory techniques used in the main paper. The electronic spin-mapping variables, which specify the electronic state of the system, are related to the electronic wavefunction coefficents for two-state systems as follows

$$S_x = 2\text{Re}[c_+^* c_-], \tag{S7a}$$

$$S_y = 2\text{Im}[c_+^* c_-], \tag{S7b}$$

$$S_z = |c_+|^2 - |c_-|^2. \tag{S7c}$$

The spin-mapping phase space that corresponds to correctly normalized electronic wavefunctions is the surface of the Bloch sphere, which satisfies $|\boldsymbol{S}| = \sqrt{S_x^2 + S_y^2 + S_z^2} = 1$. We therefore specify the following phase-space averages over the electronic and nuclear variables

$$\langle\cdots\rangle_{\text{FL}} = \frac{1}{2\pi}\int \mathrm{d}\boldsymbol{q}\int \mathrm{d}\boldsymbol{p}\int \mathrm{d}\boldsymbol{S}\,\delta(|\boldsymbol{S}|-1)\,\cdots, \tag{S8a}$$

$$\langle\cdots\rangle_{\text{PL}} = \frac{1}{(2\pi)^2}\int \mathrm{d}\boldsymbol{q}\int \mathrm{d}\boldsymbol{p}\int \mathrm{d}\boldsymbol{S}^{(\text{f})}\int \mathrm{d}\boldsymbol{S}^{(\text{b})}\,\delta\Big(|\boldsymbol{S}^{(\text{f})}|-1\Big)\,\delta\Big(|\boldsymbol{S}^{(\text{b})}|-1\Big)\,\cdots, \tag{S8b}$$

which are appropriate for both fully-linearized (FL) and partially-linearized (PL) approaches, containing one and two sets of spin-mapping variables respectively. Additionally, $\delta(x)$ is the Dirac delta function.

Surface-Hopping Approaches

Surface-hopping approaches are based on the adiabatic electronic representation and therefore it is convenient to propagate the electronic degrees of freedom in this representation[S3,S4]

$$\dot{S}_x = \frac{2\arcsin\left(O(\boldsymbol{q})\right)}{\delta t} S_z - 2V_z(\boldsymbol{q})S_y, \tag{S9a}$$

$$\dot{S}_y = 2V_z(\boldsymbol{q})S_x, \tag{S9b}$$

$$\dot{S}_z = -\frac{2\arcsin\left(O(\boldsymbol{q})\right)}{\delta t} S_x, \tag{S9c}$$

$$\dot{q}_j = \frac{p_j}{m}, \tag{S9d}$$

$$\dot{p}_j = \mathcal{F}_j^{\mathrm{SH}}(\boldsymbol{q}, \boldsymbol{S}), \tag{S9e}$$

where $O(\boldsymbol{q}(t)) = \langle \psi_+(\boldsymbol{q}(t)) | \psi_-(\boldsymbol{q}(t+\delta t)) \rangle$ is the off-diagonal element of the 2×2 overlap matrix and δt is the timestep. The advantage of using the overlap matrix rather than the NACVs for the electronic propagation is that trivial crossings between two adiabatic states are much better accounted for in the former.

Surface-hopping approaches propagate the nuclei on a single adiabatic surface at any given time. The propagation surface, n_{act}, is referred to as the 'active surface' and for the two-state system considered here, we refer to $n_{\mathrm{act}} = 1$ as the upper surface and $n_{\mathrm{act}} = -1$ as the lower. The nuclear force for surface-hopping approaches is therefore given by

$$\mathcal{F}_j^{\mathrm{SH}}(\boldsymbol{q}, \boldsymbol{S}) = -\frac{\partial \bar{V}(\boldsymbol{q})}{\partial q_j} - \frac{\partial V_z(\boldsymbol{q})}{\partial q_j} n_{\mathrm{act}}. \tag{S10}$$

In order to describe nonadiabatic transitions, the active surface must be dynamically altered in a way that mimics the dynamics of the underlying electronic wavefunction. How this is achieved depends on the specifics of the method.

The most accurate way of measuring electronic observables with a surface-hopping approach is to determine the electronic coherences with the electronic wavefunction and the adiabatic populations

using the active surface

$$\sigma_j^{\mathrm{SH}}(\boldsymbol{S}, n_{\mathrm{act}}) = \begin{cases} S_j & \text{for} \quad j \neq z, \\ n_{\mathrm{act}} & \text{for} \quad j = z, \end{cases} \tag{S11a}$$

$$\mathcal{I}^{\mathrm{SH}}(\boldsymbol{S}, n_{\mathrm{act}}) = 1, \tag{S11b}$$

which corresponds to the density matrix approach for calculating dynamical observables.[S5,S6]

Fewest-Switches Surface Hopping (FSSH)

Fewest-switches surface hopping (FSSH)[S6,S7] approximates the correlation function associated with the initial density matrix $\hat{\rho}$ [Eq. S5] and the observable $\hat{B}$ at time t as

$$\mathrm{Tr}\left[\hat{\rho}\mathrm{e}^{i\hat{H}t}\hat{B}\mathrm{e}^{-i\hat{H}t}\right] \approx \sum_{n_{\mathrm{act}}=\pm 1} \mathrm{p}_{n_{\mathrm{act}}} \left\langle \rho_{\mathrm{nuc}}(\boldsymbol{q},\boldsymbol{p})\mathcal{P}_{\rho_{\mathrm{el}}}(\boldsymbol{S})B^{\mathrm{SH}}(\boldsymbol{S}(t), n_{\mathrm{act}}(t))\right\rangle_{\mathrm{FL}}, \tag{S12}$$

where $\rho_{\mathrm{nuc}}(\boldsymbol{q},\boldsymbol{p})$ is the Wigner distribution of the initial nuclear density [Eq. S5c] and $\mathcal{P}_{\rho_{\mathrm{el}}}(\boldsymbol{S})$ is a projector onto a single point of the Bloch sphere that corresponds to the initial electronic density matrix, $\hat{\rho}_{\mathrm{el}}$

$$\mathcal{P}_{\rho_{\mathrm{el}}}(\boldsymbol{S}) = \delta(S_x - A_x(\boldsymbol{q}))\,\delta(S_y)\,\delta(S_z - A_z(\boldsymbol{q}))\,. \tag{S13}$$

This Bloch sphere point is shown schematically in the bottom-left panel of Fig. 2 of the main paper. Additionally, the initial active surface for each trajectory is determined stochastically from the initial electronic wavefunction probabilities, $\mathrm{p}_{\pm} = \frac{1}{2}(1 \pm A_z(\boldsymbol{q}))$.

In order to describe nonadiabatic transitions, the active surface is changed stochastically along the trajectory with a 'hopping probability' given by the rate of change of the underlying electronic wavefunction

$$P_{\pm\to\mp} = \pm\frac{2O(\boldsymbol{q})Sx}{1 \pm S_z}, \tag{S14}$$

where negative probabilities are set to zero and the time-step, δt, is chosen to be small enough so that the probabilities are never greater than one. In the case of a successful hop, the nuclear momentum is rescaled along the nonadiabatic coupling vector (NACV) in order to conserve energy and reflected in the case of a frustrated hop.

The Mapping Approach to Surface Hopping (MASH)

The mapping approach to surface hopping (MASH)[S2] combines the best of both worlds of FSSH and mapping-based approaches. Unlike other mapping-based approaches, MASH has a surface-hopping force akin to FSSH. The active surface in MASH is not an additional parameter within the theory, but it is determined uniquely from the spin-mapping variables. For two-state systems

$$n_{\mathrm{act}} = \mathrm{sgn}(S_z). \tag{S15}$$

This means that MASH has deterministic dynamics for which the electronic wavefunction and the active surface remain consistent throughout the dynamics. 'Hops' therefore occur in MASH every time the spin vector evolves from one hemisphere to the other. One additional advantage of MASH over FSSH is that the underlying theory uniquely prescribes how the momentum rescaling should be applied at a hop, which corresponds to rescaling along the NACV and reflecting in the case of a frustrated hop.

In order for the 'hopping times' to differ for each trajectory, the initial spin-mapping variables must be sampled. To describe an initial electronic coherence, the spin-mapping variables are sampled over the entire Bloch sphere, weighted according to

$$\mathrm{Tr}\left[\hat{\rho}\mathrm{e}^{i\hat{H}t}\hat{B}\mathrm{e}^{-i\hat{H}t}\right] \approx \tfrac{1}{2}\left\langle \rho_{\mathrm{nuc}}(\boldsymbol{q},\boldsymbol{p})\left[\mathcal{W}_{\mathrm{P}B}(\boldsymbol{S})\left(1 + A_z(\boldsymbol{q})\,\mathrm{sgn}(S_z)\right) + \mathcal{W}_{\mathrm{C}B}(\boldsymbol{S})A_x(\boldsymbol{q})\,S_x\right] B^{\mathrm{SH}}(\boldsymbol{S}(t))\right\rangle_{\mathrm{FL}}, \tag{S16}$$

The weighting factors, $\mathcal{W}_{AB}(\boldsymbol{S})$, are determined so that MASH both reproduces the short-time limit of exact quantum dynamics and the Landau-Zener transition rate in the fast nuclei limit

$$\mathcal{W}_{AB}(\boldsymbol{S}) = \begin{cases} 3 & \text{when } A = B = \mathrm{C}, \\ 2 & \text{when } A = \mathrm{P},\ B = \mathrm{C} \text{ or } A = \mathrm{C},\ B = \mathrm{P} \\ 2|S_z| & \text{when } A = B = \mathrm{P}. \end{cases} \tag{S17}$$

where C refers to a coherence operator (i.e., $\hat{\sigma}_x(\boldsymbol{q})$ and $\hat{\sigma}_y(\boldsymbol{q})$) and P to a population operator (i.e., $\hat{\mathcal{I}}$, $\hat{\sigma}_z(\boldsymbol{q})$, or the adiabatic population operators themselves).

Mean-Field Approaches

For mean-field approaches, the form of the equations of motion are invariant to the electronic representation used. However, it is often convenient to propagate the electronic degrees of freedom directly in the diabatic representation. This can be achieved by first transforming the initial adiabatic spin-mapping variables into the diabatic basis

$$S_x^{\text{diab}} = \frac{\kappa(\boldsymbol{q})S_x + \Delta(\boldsymbol{q})S_z}{V_z(\boldsymbol{q})}, \tag{S18a}$$

$$S_y^{\text{diab}} = S_y, \tag{S18b}$$

$$S_z^{\text{diab}} = \frac{-\Delta(\boldsymbol{q})S_x + \kappa(\boldsymbol{q})S_z}{V_z(\boldsymbol{q})}. \tag{S18c}$$

Then the diabatic spin variables can be propagated using the appropriate equations of motion

$$\dot{S}_x^{\text{diab}} = -2\kappa(\boldsymbol{q})S_y^{\text{diab}}, \tag{S19a}$$

$$\dot{S}_y^{\text{diab}} = 2\kappa(\boldsymbol{q})S_x^{\text{diab}} - 2\Delta(\boldsymbol{q})S_z^{\text{diab}}, \tag{S19b}$$

$$\dot{S}_z^{\text{diab}} = 2\Delta(\boldsymbol{q})S_y^{\text{diab}}, \tag{S19c}$$

$$\dot{q}_j = \frac{p_j}{m}, \tag{S19d}$$

$$\dot{p}_j = \mathcal{F}_j^{\text{MF}}(\boldsymbol{q}, \boldsymbol{S}^{\text{diab}}). \tag{S19e}$$

When required, the diabatic spin-mapping variables can then be transformed back into the adiabatic basis

$$S_x = \frac{\kappa(\boldsymbol{q})S_x^{\text{diab}} - \Delta(\boldsymbol{q})S_z^{\text{diab}}}{V_z(\boldsymbol{q})}, \tag{S20a}$$

$$S_y = S_y^{\text{diab}}, \tag{S20b}$$

$$S_z = \frac{\Delta(\boldsymbol{q})S_x^{\text{diab}} + \kappa(\boldsymbol{q})S_z^{\text{diab}}}{V_z(\boldsymbol{q})}, \tag{S20c}$$

as would be convenient for calculating electronic observables expressed using the adiabatic representation, for example.

For mean-field approaches, the nuclei are propagated according to the mean-field force, which corresponds to the expectation value of the nuclear force operator with respect to the electronic

wavefunction. In the diabatic basis, this corresponds to

$$\mathcal{F}_j^{\mathrm{MF}}(\boldsymbol{q},\boldsymbol{S}^{\mathrm{diab}}) = -\frac{\partial \bar{V}(\boldsymbol{q})}{\partial q_j} - r_{\mathrm{s}}\left[\frac{\partial \kappa(\boldsymbol{q})}{\partial q_j}S_z^{\mathrm{diab}} + \frac{\partial \Delta(\boldsymbol{q})}{\partial q_j}S_x^{\mathrm{diab}}\right], \tag{S21}$$

where r_{s} is the radius of the spin sphere.

The mean-field representation of the adiabatic Pauli spin matrices for constructing electronic observables are given in terms of the adiabatic spin-mapping variables as

$$\sigma_j^{\mathrm{MF}}(\boldsymbol{S}) = r_{\mathrm{s}}S_j. \tag{S22}$$

In addition, for Ehrenfest dynamics and spin-LSC, $\mathcal{I}(\boldsymbol{S}) = 1$, while for spin-PLDM, $\mathcal{I}(\boldsymbol{S}) = \mathcal{I}^{\mathrm{obs}}$, which is defined in Eq. S30 below.

Ehrenfest

In Ehrenfest dynamics,[S7] the electronic spin-mapping variables are initialized on the same point on the Bloch sphere ($r_{\mathrm{s}} = 1$) as in FSSH

$$\mathrm{Tr}\left[\hat{\rho}\mathrm{e}^{i\hat{H}t}\hat{B}\mathrm{e}^{-i\hat{H}t}\right] \approx \left\langle \rho_{\mathrm{nuc}}(\boldsymbol{q},\boldsymbol{p})\mathcal{P}_{\rho_{\mathrm{el}}}(\boldsymbol{S})B^{\mathrm{MF}}(\boldsymbol{S}(t))\right\rangle_{\mathrm{FL}}, \tag{S23}$$

where $\mathcal{P}_{\rho_{\mathrm{el}}}(\boldsymbol{S})$ is given by Eq. (S13).

Spin-LSC

In spin-LSC,[S8,S9] the improved accuracy over Ehrenfest dynamics arises from introducing an initial sampling over the spin sphere, even when the dynamics correspond to starting in a pure electronic state. This is achieved by using a larger spin sphere ($r_{\mathrm{s}} = \sqrt{3}$), so that

$$\mathrm{Tr}\left[\hat{\rho}\mathrm{e}^{i\hat{H}t}\hat{B}\mathrm{e}^{-i\hat{H}t}\right] \approx \left\langle \rho_{\mathrm{nuc}}(\boldsymbol{q},\boldsymbol{p})\mathcal{P}_{\mathrm{LSC}}(\boldsymbol{S})\left[1+\sqrt{3}A_z(\boldsymbol{q})S_z+\sqrt{3}A_x(\boldsymbol{q})S_x\right]B^{\mathrm{MF}}(\boldsymbol{S}(t))\right\rangle_{\mathrm{FL}}. \tag{S24}$$

The spin-LSC representation of the correlation function has the same structure as that for Ehrenfest dynamics [Eq. S23], because $\frac{1}{2}\mathcal{P}_{\rho_{\mathrm{el}}}(\boldsymbol{S})\left[1+A_z(\boldsymbol{q})S_z+A_x(\boldsymbol{q})S_x\right] = \mathcal{P}_{\rho_{\mathrm{el}}}(\boldsymbol{S})$.

Within spin-LSC, there are different options for the initial sampling of the spin-mapping vari-

ables. We choose to use so-called focused initial conditions, which sample the spin-mapping variables from the polar circles associated with the upper and lower hemispheres

$$\mathcal{P}_{\rm LSC}(\boldsymbol{S}) = \frac{1}{2}\left[\delta\left(S_z - \frac{1}{\sqrt{3}}\right) + \delta\left(S_z + \frac{1}{\sqrt{3}}\right)\right]. \tag{S25}$$

These polar circles are shown schematically by the upper-middle panel of Fig. 2 of the main paper. By using such focused initial conditions, spin-LSC is guaranteed to reproduce the correct classical nuclear dynamics on a single surface in the Born-Oppenheimer limit while also incorporating the correct description of the quantum uncertainty between the spin degrees of freedom.

Spin-PLDM

All of the methods introduced so far are so-called fully-linearized approaches,[S10,S11] which use a single set of spin-mapping variables to represent the single-time correlation function. Another class of quasiclassical methods, referred to as partially-linearized approaches,[S12–S15] use separate spin-mapping variables to represent the forward and backward time propagators within the correlation function. The partially linearized version of spin-LSC is spin-PLDM,[S16,S17] which represents the correlation function as follows

$$\mathrm{Tr}\left[\hat{\rho}\mathrm{e}^{i\hat{H}t}\hat{B}\mathrm{e}^{-i\hat{H}t}\right] \approx \left\langle \rho_{\rm nuc}(\boldsymbol{q},\boldsymbol{p})\mathcal{P}_{\rm LSC}(\boldsymbol{S}^{(\rm f)})\mathcal{P}_{\rm LSC}(\boldsymbol{S}^{(\rm b)})B^{\rm MF}(\boldsymbol{S}^{\rm obs}(t))\right\rangle_{\rm PL}, \tag{S26}$$

where $\boldsymbol{S}^{(\rm f)}$ and $\boldsymbol{S}^{(\rm b)}$ are the spin-mapping variables for the forward and backward propagation respectively. These mapping variables are sampled from the same polar circles as in spin-LSC (Eq. S25), which means that the centroid, $\boldsymbol{S} = \frac{1}{2}(\boldsymbol{S}^{(\rm f)} + \boldsymbol{S}^{(\rm b)})$ is distributed according to the upper-right panel of Fig. 2 of the main paper. In spin-PLDM, the centroid is the dynamical variable that determines the nuclear force (via Eqs. (S9) and (S21) in the diabatic basis with $r_{\rm s} = \sqrt{3}$).

In addition, the observables in spin-PLDM are determined by another set of spin-mapping variables, $\boldsymbol{S}^{\rm obs}$, related to $\boldsymbol{S}^{(\rm f)}$ and $\boldsymbol{S}^{(\rm b)}$ by

$$S_j^{\rm obs}(t) = \frac{4}{\sqrt{3}}\mathrm{Re}\left[\mathrm{tr}\left[\hat{\rho}_{\rm el}\hat{w}\left(\boldsymbol{S}^{(\rm b)}\right)\hat{U}^{\dagger}(t)\hat{\sigma}_j(\boldsymbol{q}(t))\hat{U}(t)\hat{w}\left(\boldsymbol{S}^{(\rm f)}\right)\right]\right]. \tag{S27}$$

Here $\mathrm{tr}[\cdots]$ refers to the quantum trace over the electronic degrees of freedom, $\hat{U}$ is the time-ordered

electronic propagator along the nuclear path and $\hat{w}(\boldsymbol{S})$ is the Stratonovich-Weyl kernel given by

$$\hat{w}(\boldsymbol{S}) = \frac{1}{2}\left[\hat{\mathcal{I}} + \sqrt{3}\boldsymbol{S}\cdot\hat{\boldsymbol{\sigma}}(q)\right]. \tag{S28}$$

While Eq. (S27) is in principle sufficient for calculating $\boldsymbol{S}^{\text{obs}}(t)$, we propose an easier scheme in practice for propagating these spin-mapping variables along the trajectory. Firstly from Eq. (S27), the $t = 0$ values of $\boldsymbol{S}^{\text{obs}}$ can be determined as follows

$$S_x^{\text{obs}}(0) = \left(S_x^{(\text{f})} + S_x^{(\text{b})}\right) + \sqrt{3}A_z(\boldsymbol{q})\left(S_z^{(\text{f})}S_x^{(\text{b})} + S_z^{(\text{b})}S_x^{(\text{f})}\right) + \sqrt{3}A_x(\boldsymbol{q})\left(2S_x^{(\text{f})}S_x^{(\text{b})} + \frac{1}{3} - \boldsymbol{S}^{(\text{f})}\cdot\boldsymbol{S}^{(\text{b})}\right), \tag{S29a}$$

$$S_y^{\text{obs}}(0) = \left(S_y^{(\text{f})} + S_y^{(\text{b})}\right) + \sqrt{3}A_z(\boldsymbol{q})\left(S_z^{(\text{f})}S_y^{(\text{b})} + S_z^{(\text{b})}S_y^{(\text{f})}\right) + \sqrt{3}A_x(\boldsymbol{q})\left(S_x^{(\text{f})}S_y^{(\text{b})} + S_x^{(\text{b})}S_y^{(\text{f})}\right), \tag{S29b}$$

$$S_z^{\text{obs}}(0) = \left(S_z^{(\text{f})} + S_z^{(\text{b})}\right) + \sqrt{3}A_z(\boldsymbol{q})\left(2S_z^{(\text{f})}S_z^{(\text{b})} + \frac{1}{3} - \boldsymbol{S}^{(\text{f})}\cdot\boldsymbol{S}^{(\text{b})}\right) + \sqrt{3}A_x(\boldsymbol{q})\left(S_x^{(\text{f})}S_z^{(\text{b})} + S_x^{(\text{b})}S_z^{(\text{f})}\right). \tag{S29c}$$

The initial values of the observable spin-mapping variables, $\boldsymbol{S}^{\text{obs}}(0)$, can be transformed into the diabatic basis using Eq. (S18) and then propagated according to Eqs. (S19a), (S19b) and (S19c) along the original nuclear trajectory. At each timestep, the observable spin-mapping variables in the diabatic basis could also be transformed back to the adiabatic basis with Eq. (S20) if desired.

Finally, noting that $\hat{U}^\dagger(t)\hat{U}(t) = \hat{\mathcal{I}}$, the spin-PLDM representation of the identity operator is given by

$$\begin{aligned}\mathcal{I}^{\text{obs}} &= 4\text{Re}\left[\text{tr}\left[\hat{\rho}_{\text{el}}\hat{w}\left(\boldsymbol{S}^{(\text{b})}\right)\hat{w}\left(\boldsymbol{S}^{(\text{f})}\right)\right]\right] \\ &= \left(1 + 3\boldsymbol{S}^{(\text{f})}\cdot\boldsymbol{S}^{(\text{b})}\right) + \sqrt{3}A_z(\boldsymbol{q})\left(S_z^{(\text{f})} + S_z^{(\text{b})}\right) + \sqrt{3}A_x(\boldsymbol{q})\left(S_x^{(\text{f})} + S_x^{(\text{b})}\right).\end{aligned} \tag{S30}$$

Evaluation of the Coherence Measures

In order to evaluate the coherence measures introduced in the main paper with quasiclassical trajectory approaches, the trajectories are first used to compute the correlation functions associated with $\hat{B} = \hat{\sigma}_x(\boldsymbol{q})$, $\hat{\sigma}_y(\boldsymbol{q})$, $\hat{\sigma}_x(\boldsymbol{q})\,|\boldsymbol{q}\rangle\langle\boldsymbol{q}|$ and $\hat{\sigma}_y(\boldsymbol{q})\,|\boldsymbol{q}\rangle\langle\boldsymbol{q}|$. The coherence measures are then constructed in terms of the correlation functions using the formulas provided in the main paper.

In order to calculate the correlation functions involving the nuclear projection operator, $\hat{P}_{\boldsymbol{q}} = |\boldsymbol{q}\rangle\langle\boldsymbol{q}|$, we note that the Wigner transform of this quantity is

$$P_{\boldsymbol{q}}^{\mathrm{W}}(\boldsymbol{q}'(t),\boldsymbol{p}'(t)) = \delta(\boldsymbol{q}-\boldsymbol{q}'(t)). \tag{S31}$$

This can be evaluated by histogramming the trajectories.

For the numerical simulations, 10^6 trajectories were used with a timestep of 0.1 fs. All trajectories of the mapping-based approaches were repeated once with the initial conditions switched as follows: $S_x \to -S_x$ and $S_y \to -S_y$. For spin-PLDM, this replacement was performed independently for $\boldsymbol{S}^{(\mathrm{b})}$ and $\boldsymbol{S}^{(\mathrm{f})}$, meaning that the trajectories were actually repeated four times in this case. The $\mathcal{C}_{-+}(t)$ coherence measure and the nuclear coordinate density were computed using equally spaced bins of width $\Delta q = 0.005$ between $q = -7$ and $q = 7$. A large number of trajectories were used to make sure that the $\mathcal{C}_{-+}(t)$ coherence measure was sufficiently converged for such a fine grid. Far fewer trajectories would have been needed to compute the other observables, as well as this coherence measure on a coarser grid.

The Models

One-Dimensional Model

We consider the same one-dimensional model used in Refs. S1,S18. This is the main model considered throughout the main paper. The diabatic Hamiltonian parameters in atomic units are

$$\bar{V}(q) = \tfrac{1}{2}kq^2, \tag{S32a}$$

$$\Delta(q) = b\mathrm{e}^{-a\left(q-\frac{\epsilon}{2k\bar{q}}\right)^2}, \tag{S32b}$$

$$\kappa(q) = k\bar{q}q - \tfrac{1}{2}\epsilon, \tag{S32c}$$

where $k = 0.02$, $b = 0.01$, $a = 3$, $\bar{q} = -2$ and $\epsilon = 0.01$. The quantities used here are related to those used in Refs. S1,S18 by: $q = R - \frac{1}{2}(R_1 + R_2)$ and $\bar{q} = \frac{1}{2}(R_2 - R_1)$. Additionally, $m = 20000$.

Figure S1 gives the coherence measures calculated for this model in the Born-Oppenheimer

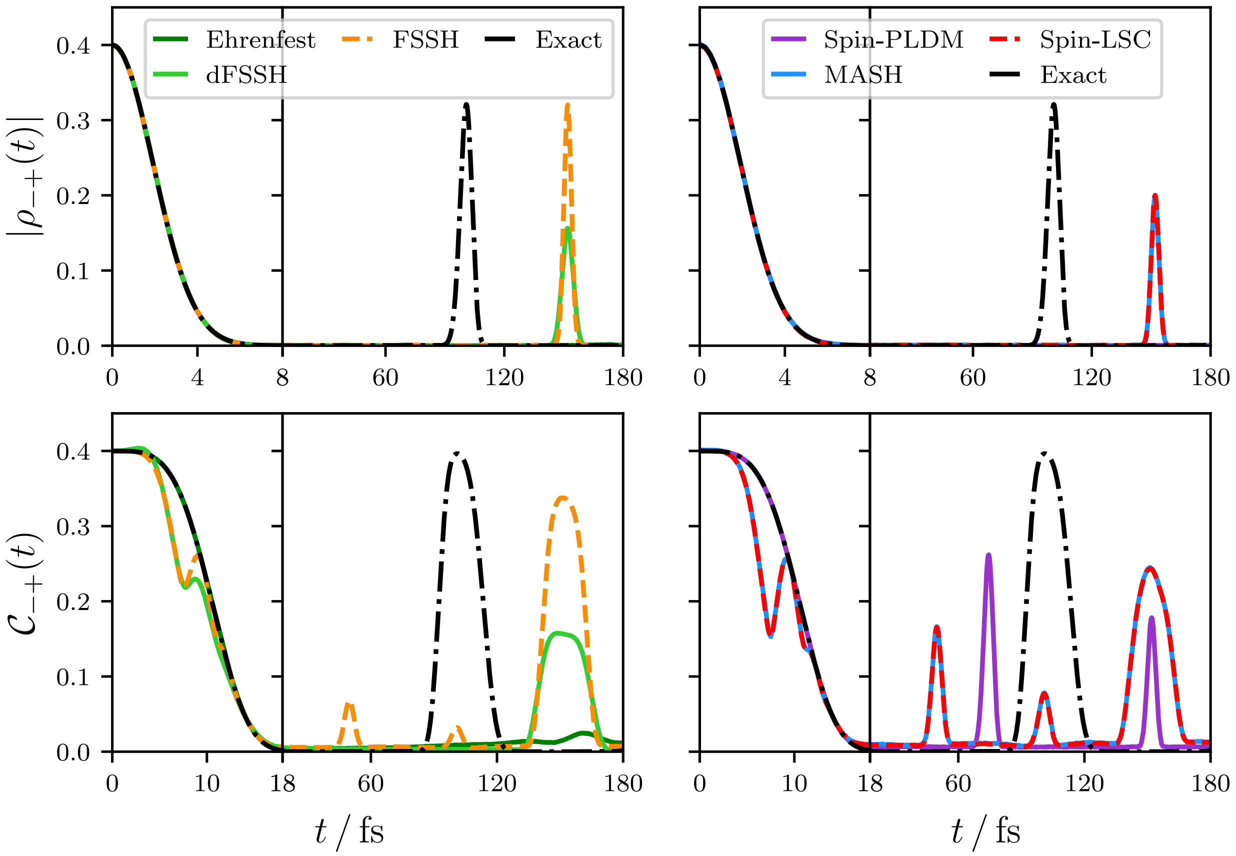

Figure S1: The Born-Oppenheimer limit of the coherence measures shown in Fig. 5 of the main paper.

limit (the same quantities associated with the full nonadiabatic dynamics are given in Fig. 5 of the main paper). The Born-Oppenheimer limit corresponds to setting the NACV (and the off-diagonal element of the overlap matrix) in the equations of motion to zero.

BMA Model

In order to give a more realistic description of photoexcited processes in molecules we also consider the bis(methylene) adamantyl cation (BMA), using a two-dimensional conical intersection model.[S19]

The form of this conical intersection model is given in atomic units by

$$\bar{V}(\boldsymbol{q}) = \tfrac{1}{2}\left(\omega_x^2 q_x^2 + \omega_y^2 q_y^2\right), \tag{S33a}$$

$$\Delta(\boldsymbol{q}) = c q_y, \tag{S33b}$$

$$\kappa(\boldsymbol{q}) = \frac{1}{2}\omega_x^2 a q_x, \tag{S33c}$$

where $\omega_x = 7.743 \times 10^{-3}$ and $\omega_y = 6.68 \times 10^{-3}$ are the vibrational frequencies of the two nuclear modes given in mass-weighted coordinates (so that effectively $m = 1$), such that the corresponding spring constants are given by $k_j = \omega_j^2$. Additionally, $a = 31.05$ and $c = 8.092 \times 10^{-5}$. Finally, the initial Gaussian wavepacket is centered at the ground-state equilibrium geometry of the cation, given by $\bar{q}_x = \frac{a}{2} = 15.525$ and $\bar{q}_y = 0$.

References

(S1) Villaseco Arribas, E.; Maitra, N. T.; Agostini, F. Nonadiabatic dynamics with classical trajectories: The problem of an initial coherent superposition of electronic states. *J. Chem. Phys.* **2024**, *160*, 054102.

(S2) Mannouch, J. R.; Richardson, J. O. A mapping approach to surface hopping. *J. Chem. Phys.* **2023**, *158*, 104111.

(S3) Jain, A.; Alguire, E.; Subotnik, J. E. An Efficient, Augmented Surface Hopping Algorithm That Includes Decoherence for Use in Large-Scale Simulations. *J. Chem. Theory Comput.* **2016**, *12*, 5256–5268.

(S4) Lawrence, J. E.; Ansari, I. M.; Mannouch, J. R.; Manae, M. A.; Asnaashari, K.; Kelly, A.; Richardson, J. O. A MASH simulation of the photoexcited dynamics of cyclobutanone. *J. Chem. Phys.* **2024**, *160*, 174306.

(S5) Landry, B. R.; Falk, M. J.; Subotnik, J. E. Communication: The correct interpretation of surface hopping trajectories: How to calculate electronic properties. *J. Chem. Phys.* **2013**, *139*, 214107.

(S6) Subotnik, J. E.; Jain, A.; Landry, B.; Petit, A.; Ouyang, W.; Bellonzi, N. Understanding the surface hopping view of electronic transitions and decoherence. *Annu. Rev. Phys. Chem.* **2016**, *67*, 387–417.

(S7) Tully, J. C. Molecular dynamics with electronic transitions. *J. Chem. Phys.* **1990**, *93*, 1061–1071.

(S8) Runeson, J. E.; Richardson, J. O. Spin-mapping approach for nonadiabatic molecular dynamics. *J. Chem. Phys.* **2019**, *151*, 044119.

(S9) Runeson, J. E.; Richardson, J. O. Generalized spin mapping for quantum-classical dynamics. *J. Chem. Phys.* **2020**, *152*, 084110.

(S10) Miller, W. H. The Semiclassical Initial Value Representation: A Potentially Practical Way

for Adding Quantum Effects to Classical Molecular Dynamics Simulations. *J. Phys. Chem. A* **2001**, *105*, 2942–2955.

(S11) Gao, X.; Saller, M. A. C.; Liu, Y.; Kelly, A.; Richardson, J. O.; Geva, E. Benchmarking Quasiclassical Mapping Hamiltonian Methods for Simulating Electronically Nonadiabatic Molecular Dynamics. *J. Chem. Theory Comput.* **2020**, *16*, 2883–2895.

(S12) Huo, P.; Coker, D. F. Communication: Partial linearized density matrix dynamics for dissipative, non-adiabatic quantum evolution. *J. Chem. Phys.* **2011**, *135*, 201101.

(S13) Huo, P.; Coker, D. F. Consistent schemes for non-adiabatic dynamics derived from partial linearized density matrix propagation. *J. Chem. Phys.* **2012**, *137*, 22A535.

(S14) Hsieh, C.-Y.; Kapral, R. Nonadiabatic dynamics in open quantum-classical systems: Forward-backward trajectory solution. *J. Chem. Phys.* **2012**, *137*, 22A507.

(S15) Hsieh, C.-Y.; Kapral, R. Analysis of the forward-backward trajectory solution for the mixed quantum-classical Liouville equation. *J. Chem. Phys.* **2013**, *138*, 134110.

(S16) Mannouch, J. R.; Richardson, J. O. A partially linearized spin-mapping approach for nonadiabatic dynamics. I. Derivation of the theory. *J. Chem. Phys.* **2020**, *153*, 194109.

(S17) Mannouch, J. R.; Richardson, J. O. A partially linearized spin-mapping approach for nonadiabatic dynamics. II. Analysis and comparison with related approaches. *J. Chem. Phys.* **2020**, *153*, 194110.

(S18) Pieroni, C.; Agostini, F. Nonadiabatic Dynamics with Coupled Trajectories. *J. Chem. Theory Comput.* **2021**, *17*, 5969–5991.

(S19) Ryabinkin, I. G.; Joubert-Doriol, L.; Izmaylov, A. F. When do we need to account for the geometric phase in excited state dynamics? *J. Chem. Phys.* **2014**, *140*, 214116.
